# Spatiotemporal Analysis of Guaroa Virus Diversity, Evolution, and Spread in South America

**DOI:** 10.3201/eid2103.141351

**Published:** 2015-03

**Authors:** Allison Groseth, Kurt R. Wollenberg, Veena Mampilli, Taylor Shupert, Carla Weisend, Carolina Guevara, Tadeusz J. Kochel, Robert B. Tesh, Hideki Ebihara

**Affiliations:** National Institutes of Health, Hamilton, Montana, USA (A. Groseth, V. Mampilli, T. Shupert, C. Weisend, H. Ebihara);; National Institutes of Health, Bethesda Maryland, USA (K.R. Wollenberg);; US Naval Medical Research Unit-6, Lima, Peru (C. Guevara, T.J. Kochel);; University of Texas Medical Branch, Galveston, Texas, USA (R.B. Tesh)

**Keywords:** bunyavirus, orthobunyavirus, genetic characterization, genetic diversity, genetic evolution, phylogeny, phylogeography, spatiotemporal analysis, Guaroa virus, virus spread, viruses, South America

## Abstract

We conducted phylogeographic modeling to determine the introduction and spread of Guaroa virus in South America. The results suggest a recent introduction of this virus into regions of Peru and Bolivia over the past 60–70 years and emphasize the need for increased surveillance in surrounding areas.

Guaroa virus (GROV; family *Bunyaviridae*, genus *Orthobunyavirus*) infection in humans frequently results in febrile illness, and limited serologic surveillance indicates that the virus infects a substantial portion of the rural population in tropical regions of Central and South America (Figure 1, panel A) (*1*,*2*). However, similar to the situation for many arboviruses found in the Neotropics, limited systematic surveillance for GROV in rural communities contributes to a lack of information regarding the ecology and actual effect of GROV on public health. In addition, the very limited resources available for such work results in a critical need for approaches that can help identify where these resources can be put to best use. To further our knowledge of GROV in South America, we conducted a spatiotemporal analysis to estimate when and where the virus was introduced into the region, model its subsequent pattern of spread, and obtain insights into the ecologic and anthropogenic factors that may have affected these processes.

**Figure 1:**
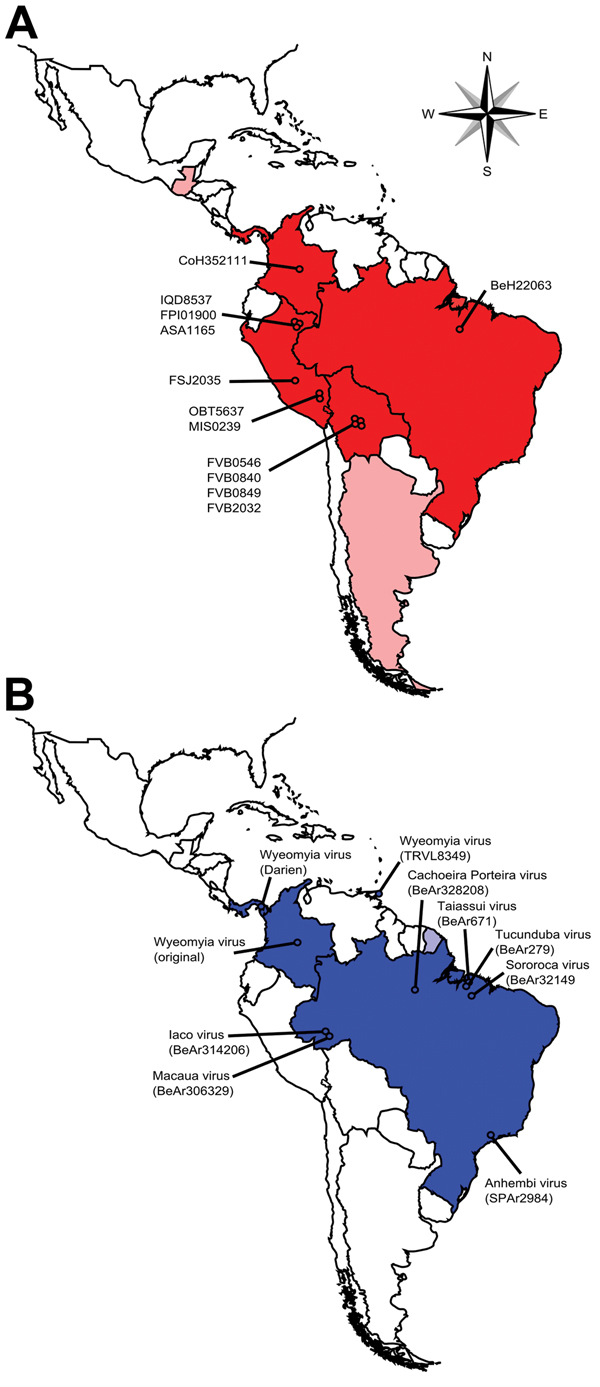
Comparison of the geographic locations from which Guaroa virus (GROV) strains used in this study were isolated (A) and the geographic distribution of the Wyeomyia virus (WYOV) group (B), Central and South America. Countries from which GROVs have been isolated are shown in dark red; countries from which WYOVs have been isolated are shown in dark blue. Light red indicates countries with only serologic evidence of GROV circulation; light blue indicates countries with only serologic evidence of WYOV circulation. Circles indicate the geographic locations from which the virus strains used in the present study were isolated.

## The Study

To generate the large, complete genome dataset needed for phylogeographic analysis, we obtained 12 GROV strains from the American Type Culture Collection (ATTC VR-394, strain CoH352111; Manassas, VA, USA) or from the World Reference Center for Emerging Viruses and Arboviruses (all other GROV strains; University of Texas Medical Branch, Galveston, TX, USA). These viruses had been isolated during 1956–2011 and originate from 4 of the 5 countries in which GROV has been isolated: Columbia, Brazil, Peru, and Bolivia (Figure 1, panel A; Technical Appendix Table). Clinical specimens used in this study were obtained under the terms of a human use protocol (NMRCD.2000.0006). This protocol and the consent procedure were approved by the Naval Medical Research Center Institutional Review Board in compliance with all US and Peruvian federal regulations governing the protection of human subjects. 

Viral RNA was extracted from the isolates by using the QIAamp Viral RNA Mini Kit (QIAGEN, Valencia, CA, USA), and reverse transcription PCR reactions were performed by using the Superscript III Reverse Transcriptase (Life Technologies, Grand Island, NY, USA) and the iProof High-Fidelity PCR Kit (Bio-Rad, Hercules, CA, USA). Coding region sequencing was based on partial sequences of strain BeH22063 (GenBank accession nos. X73466 [S segment], AY380581 [M segment], JN801039 [L segment]) and completed where necessary by using primer walking. Terminal noncoding sequences were obtained by using ligation-anchored PCR, as previously described (*3*). Complete genome sequences were deposited in GenBank (Technical Appendix Table); primer sequences are available upon request.

We then used the large GROV dataset and one for the closely related Wyeomyia virus (WYOV) group to conduct a phylogeographic analysis (*4*). GROV and WYOV are geographically restricted to Central and South America, but the next most closely related virus clades are not found in the region, suggesting a specific introduction of the GROV/WYOV common ancestor into South America (Figure 1). We calculated Bayesian coalescent phylogenies, incorporating sample times and locations (Technical Appendix Table), by using BEAST v1.8.0 (http://beast.bio.ed.ac.uk/) with multiple sequence alignments containing nucleoprotein open-reading frame sequences from the GROV and WYOV groups. The resulting trees, along with tables containing the geographic coordinates of the samples, were then input into SPREAD v1.0.6 (*5*) to calculate ancestral locations (Technical Appendix Figure) and 80% highest posterior density polygons. Graphical map overlays were generated, viewed by using Google Earth (https://www.google.com/earth), and exported as video files (Video 1 and Video 2). Because decimal coordinates were used for sample locations, we performed a continuous phylogeographic analysis (*6*).

**Video 1:**
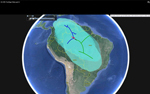
Overview, with highest posterior density polygons, of the predicted spread of Guaroa virus and Wyeomyia and Anhembi lineage viruses over time in South America.

**Video 2:**
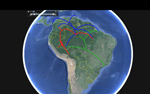
Overview, without highest posterior density polygons, of the predicted spread of Guaroa virus and Wyeomyia and Anhembi lineage viruses over time in South America.

## Conclusions

**Figure 2:**
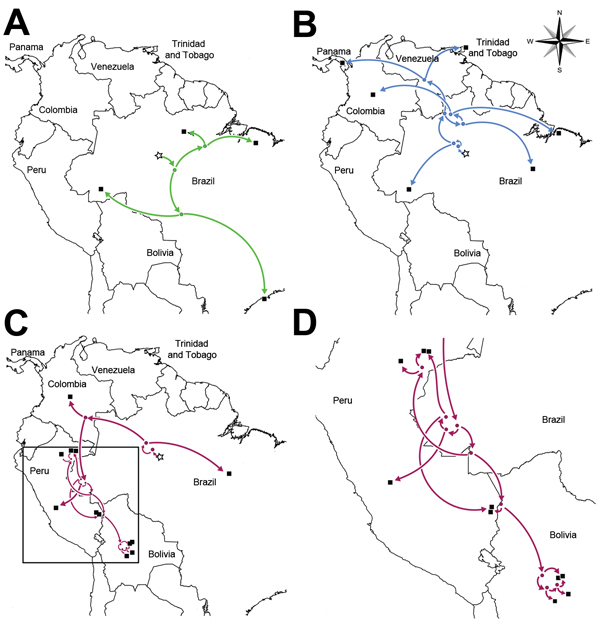
Spread (arrows) of Wyeomyia virus (WYOV) group viruses and Guaroa virus (GROV) in Central and South America. A) Anhembi lineage WYOV group viruses; B) Wyeomyia lineage WYOV group viruses; C) GROVs. D) Enlargement of boxed area in panel C, showing the spread of GROV in Bolivia and Peru, as determined by phylogeographic analysis. Bayesian coalescent phylogenies incorporating sample times and locations (Technical Appendix Table) were calculated for the nucleoprotein open-reading frame dataset by using BEAST v1.8.0 (http://beast.bio.ed.ac.uk/) and then input into SPREAD v1.0.6 (*5*) to calculate ancestral locations and corresponding graphical map overlays. Stars in each panel represent the predicted site of introduction for the GROV/WYOV common ancestor; dots represent the predicted locations associated with all other nodes (Technical Appendix Figure). Black boxes indicate the locations at which the viruses used in this study were isolated.

The results of our analysis indicate that the GROV/WYOV common ancestor was introduced into South America ≈250 years ago (i.e., about 1764) at a site along the Amazon River; the identified site is within ≈250 km of Manaus, Brazil, which has been a major population center in this area since the early days of European colonization, possibly even longer (*7*) (Figure 2; Video 1 and Video 2). This estimated date corresponds with a tumultuous period in South American history, during which various revolts, insurrections, and wars for independence were taking place. In addition, this era was associated with record levels of slave importation from Africa to facilitate the growing economy of Brazil (*8*). Thus, the GROV/WYOV common ancestor might have been introduced by several different means during the transport of persons, supplies, or both. Furthermore, our estimated date and location of introduction is consistent with that determined for the introduction of yellow fever virus (YFV) into Brazil (*9*), indicating that the geopolitical conditions at this time may in fact have facilitated the introduction and subsequent spread of several different viruses into this area.

From this central introduction point of the GROV/WYOV common ancestor, the GROV group and the WYOV group, which is further made up of 2 distinct lineages (the WYOV and Anhembi virus [ABMV] lineages), exhibit differences in their patterns of spread. The ABMV lineage spread in a predominantly southward direction within Brazil (Figure 2, panel A), and the WYOV lineage spread northward into northern Brazil and, ultimately, into Columbia, Central America, and the Caribbean (Figure 2, panel B). In contrast, although the spread of GROV initially closely followed that of the WYOV lineage into the northern regions of South America, our results indicate that over the past 60–70 years, it spread rapidly spread southward into Peru and, subsequently, into Bolivia (Figure 2, panels C and D; Video 1 and Video 2). Recent spread of GROV into the regions of Peru and Bolivia highlights the need for focused surveillance in these areas to monitor for continued spread of GROV into surrounding areas. This finding also appears to be consistent with serologic investigations in this area, which indicate that, whereas the virus has only recently been detected in persons in these regions (*2*), GROV antibody has been detected in serum samples from the population dating back to 1965 (*10*).

The observed spatial patterns are also notable in that they suggest possible mechanisms of virus spread within the endemic region. The northwest/southeast axis of spread seen with the WYOV and ABMV lineages closely reflects what was reported for YFV (*9*), which, given the nonsylvatic nature of YFV transmission in South America, suggests a direct contribution of human, mosquito, or both populations to this pattern of spread. However, this finding may not adequately explain the spread pattern observed for GROV. This pattern might be explained by the involvement of avian reservoirs: ecological data indicate the infection of South American birds with several closely related orthobunyaviruses that are endemic to the New World (*11*). Furthermore, Columbia represents an intersection point between flyways from Brazil and those that run along the western coast of South America (*12*). These observations then raise the possibility that unidentified bird species, including migratory birds, could also be involved in the spread of GROV, and suggest that avian species should be considered a priority for future surveillance efforts within the GROV-endemic region.

Overall, our study has led to the generation of a substantial set of full-length GROV sequences, which we anticipate will aid in future efforts to develop improved diagnostic approaches for this and related viruses, and has enabled us to model the spread of GROV within the virus-endemic area. In doing so, we have identified Peru and Bolivia as regions of recent and active GROV spread that should be considered areas for future virus surveillance efforts. In addition, our data implicate both human-associated factors and possibly bird populations in the spread of this virus within South America.

Technical Appendix Source and sequence information for virus strains used in this study and phylogeographic analysis of the predicted spread of Guaroa virus and Wyeomyia and Anhembi lineage viruses over time in South America.
