# Supplementary material for: Spatiotemporal Analysis of Guaroa Virus Diversity, Evolution, and Spread in South America
Source: Emerg Infect Dis. 2015 Mar;21(3):460–3. doi: 10.3201/eid2103.141351 (PMC4345823; doi:10.3201/eid2103.141351)
Supplement: Technical Appendix — Source and sequence information for virus strains used in this study and phylogeographic analysis of the predicted spread of Guaroa virus and Wyeomyia and Anhembi lineage viruses over time in South America. [file 14-1351-Techapp-s1.pdf]

Article DOI: http://dx.doi.org/10.3201/eid2103.141351

Spatiotemporal Analysis of Guaroa Virus Diversity, Evolution, and Spread in South America

Technical Appendix

Technical Appendix Table. Source and sequence information for virus strains used in a phylogeographic modeling study of the evolution, spread, and diversity of Guaroa virus in South America*

Virus, strain	Host	Year isolated	Geographic location of isolation: Locale	Geographic location of isolation: Approximate GPS coordinates	GenBank accession nos.†
Guaroa					
CoH352111	Human (asymptomatic)	1956	Guaroa, Meta Department, Colombia	3.86, −73.50	KM245519–21
BeH22063	Human	1960	Pará, Brazil	−1.45, −48.46	KM245522–4
IQD8537	Human	2004	Iquitos, Loreto, Peru	−3.76, −73.26	KM245525–7
FVB0546	Human	2007	Chapare, Cochabamba Department, Bolivia	−16.67, −65.58	KM245528–30
OBT5637	Human	2007	Puerto Maldonado, Madre de Dios, Peru	−12.59, −69.20	KM245531–3
MIS0239	Human	2008	Puerto Maldonado, Madre de Dios, Peru	−12.59, −69.20	KM245534–6
FVB0840	Human	2009	Tiraque, Cochabamba Department, Bolivia	−17.43, −65.72	KM245537–9
FVB0849	Human	2009	Chapare, Cochabamba Department, Bolivia	−16.67, −65.58	KM245540–2
FVB2032	Human	2009	Chapare, Cochabamba Department, Bolivia	−16.67, −65.58	KM245543–5
FPI01900	Human	2011	Loreto, Peru	−4.23, −74.53	KM245546–8
FSJ2035	Human	2011	Chanchamayo, Peru	−11.05, −75.1	KM245549–51
ASA1165	Human	2011	Iquitos, Loreto Peru	−3.76, −73.26	KM245552–4
Anhembi					
SPAr2984	*Phoniomyia pilicauda* mosquitos	1965	Casa Grande, São Paolo, Brazil	−23.67, −45.92	JN572064–2
Cachoeira Porteira					
BeAr328208	*Sabethes glaucodaemon* mosquitos	1977	Cachoeira Porteira, Pará, Brazil	−1.07, −57.04	JN968590–2
Iaco BeAr314206	*Wyeomyia* spp. mosquitos	1976	Sena Madureira, Acre, Brazil	−9.08, −68.67	JN572065–7
Macaua BeAr306329	*Sabethes soperi* mosquitos	1976	Sena Madureira, Acre, Brazil	−9.08, −68.67	JN572068–70
Sororoca BeAr32149	*Sabethini* spp. mosquitos	1961	Belem–Brasilia Highway (km 94), Pará, Brazil	−2.44, −47.51	JN572071–3
Taiassui BeAr671	*Sabethini* spp. mosquitos	1955	Taiassui River Basin (Benevides), Pará, Brazil	−1.37, −48.24	JN572074–6
Tucunduba virus BeAr278	*Sabethini* spp. mosquitos	1955	Belem, Pará, Brazil	−1.45, −48.46	JN572077–9
Wyeomyia virus Original	*Wyeomyia melanocephala* mosquitos	1940	Villavicencio, Meta, Columbia	4.15, −73.64	JN572080–2
Wyeomyia virus TRVL8349	*Psorophora albipes* mosquitos	1955	Melajo Forest Reserve, Trinidad	10.64, −61.08	JN801033–5

Virus, strain	Host	Year isolated	Geographic location of isolation: Locale	Geographic location of isolation: Approximate GPS coordinates	GenBank accession nos.†
Wyeomyia virus					
Darien	Human	1963	Pan American Highway, Darien Province, Panama	8.49, −77.97	JN801036–8

*GPS, Global Positioning System.
†Genomic sequences for all Guaroa virus strains were determined as a part of this study.

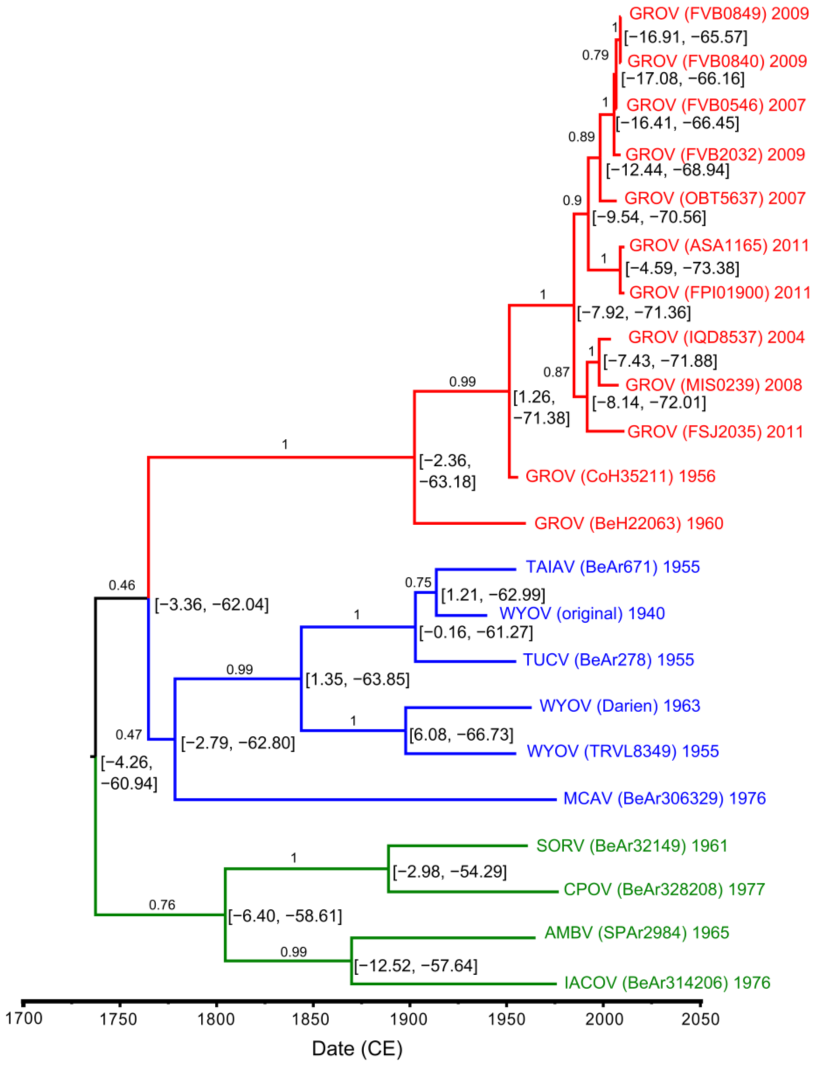

Technical Appendix Figure. Phylogeographic analysis of the spread of GROV, WYOV lineage, and AMBV lineage. Bayesian coalescent phylogenies incorporating sample times and location were calculated on the basis of the nucleoprotein open-reading frame dataset by using BEAST v1.8.0 (http://beast.bio.ed.ac.uk/) and then input into SPREAD v1.0.6 (*1*) to calculate ancestral locations for all antecedent nodes in the GROV and WYOV groups. The bracketed values at nodes represent the estimated locations of the ancestral sequences. The numbers above the branches are posterior probabilities of the subtended clades; p>0.95 represents significant support for that group. Red indicates

Guaroa viruses; blue indicates Wyeomyia group viruses (Wyeomyia lineage); and green indicates Wyeomyia group viruses (Anhembi lineage). AMBV, Anhembi virus; CPOV, Cachoeira Porteira virus; GROV, Guaroa virus; IACOV, Iaco virus; MCAV, Macaua virus; SORV, Sororoca virus; TAIAV, Taiassui virus; TUCV, Tucunduba virus; WYOV, Wyeomyia virus. Scale bar represents estimated dates for various events.

Reference

1. Bielejec F, Rambaut A, Suchard MA, Lemey P. SPREAD: spatial phylogenetic reconstruction of evolutionary dynamics. Bioinformatics. 2011;27:2910–2. PubMed http://dx.doi.org/10.1093/bioinformatics/btr481
